# Case report on managing incomplete bone formation after bilateral sinus augmentation using a palatal approach and a dilating balloon technique

**DOI:** 10.1186/s40729-017-0065-7

**Published:** 2017-01-19

**Authors:** Tobias K. Boehm

**Affiliations:** Western University of Health Sciences College of Dental Medicine, 309 E Second Street, Pomona, CA 91766 USA

**Keywords:** Maxilla, Complication, Implant

## Abstract

**Background:**

Patients with resorbed edentulous alveolar ridges in the posterior maxilla often require lateral window sinus augmentation procedures prior to implant placement. Lateral window sinus augmentation procedures can produce incomplete bone augmentation as consequence of surgical and healing complications producing unusual and complex sinus anatomy. Although incomplete bone formation after sinus augmentation has been described in a previous case reports, this is the first case report that describes grafting these compromised sites prior to implant placement.

**Case presentation:**

A 65-year-old male patient with no known medical conditions presented with severe chronic localized periodontitis and a combined periodontal-endodontic lesion affecting three first molars. Initial ridge preservation and lateral window sinus augmentation resulted in incomplete bone formation and complex sinus floor anatomy on both right and left sides. A dilating balloon technique on one side and a palatal approach on the other side were utilized for additional sinus augmentation using particulate allograft and resorbable collagen membranes. Healing was uneventful, and implants could be placed and restored at all sites. Periodontal maintenance was conducted every 3 months, and the implants have been in function and periodontally healthy for 2 years.

**Conclusion:**

Despite initial failure of sinus augmentation to produce suitable implant sites, it is possible to rescue these sites with re-entry grafting procedures and allow successful implant placement and restoration.

## Background

Patients with severe periodontal disease often display severely resorbed ridges in the posterior maxilla. Implant therapy can be a challenge for those patients as available bone height is limited by the maxillary sinus. Although sinus augmentation using subantral or lateral window approaches are routinely used, complications occur that may limit bone augmentation in the sinus after any given procedure. The most common complication during sinus augmentation surgery is tearing of the Schneiderian membrane. This happens in 14–53% of surgeries. History of tobacco use and complex sinus anatomy are the most common risk factors for membrane tears. Membrane tears that develop during the surgery can be managed by placing resorbable membranes over the torn area [1–3]. Although piezoelectric surgery and surgical planning can reduce this complication [4], tears still remain a possible surgical complication and there may be incomplete bone augmentation [5].

One reason for this is that even though piezoelectric surgery can gently remove the overlying bone from the fragile Schneiderian membrane, sinus curettes still may be needed to manually lift the membrane from the interior walls of the sinus. As this procedure can tear the membrane, Dr. Muronoi and others developed an alternative procedure for lifting the Schneiderian membrane using a hemostatic nasal dilating balloon in 2003. For this procedure, the surgeons created a lateral window in the posterior maxilla exposing the Schneiderian membrane, slightly elevate the membrane, insert a dilating balloon, and use hydraulic pressure to inflate the balloon, which then gently separates the membrane from the underlying bone and creates space for bone grafting materials [6]. Other clinicians refined this technique by creating successively smaller access windows and reported complications in less than 10% of cases, only minor patient discomfort and satisfactory bone formation [7–10]. Most recently, several clinicians modified the procedure by further reducing the flap size needed for the procedure, moving the access site to the ridge crest, and limit the access window to an implant osteotomy created with osteotomes [11, 12]. Significantly for our case report, this transcrestal approach reduces the chance of postgrafting complications with patients who have sinus pathology and unusual sinus anatomy while minimizing the chance of membrane tears [13].

Membrane tears are a significant concern as they may result in postoperative complications such as an oroantral communication as reported recently. In this case, the communication was managed by inserting a fibrin sponge, but it resulted in a cyst-like concavity within grafted bone, which was subsequently managed by re-entry and grafting of the affected site prior to implant placement [14]. As seen in this case, incomplete bone formation can be managed with re-entry procedures, but incomplete bone formation often results in unusual sinus floor morphologies that make conventional sinus approaches difficult. A recent case report describes an unconventional palatal approach for managing sinus floor anatomy complicated by previous sinus grafting [15].

There is still little data on the long-term success of these unconventional re-entry procedures after incomplete bone formation, and here, we present a case with 3-year follow-up after re-entry grafting procedures using either a palatal window or balloon-dilating device for management of previously failed sinus augmentation.

## Case presentation

**Fig. 1:**
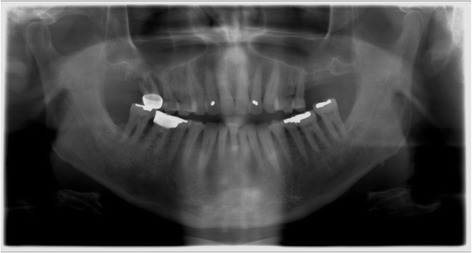
Initial presentation. Panoramic radiograph taken at initial visit shows severe bone loss, supraerupted molars and furcation involvement

**Fig. 2:**
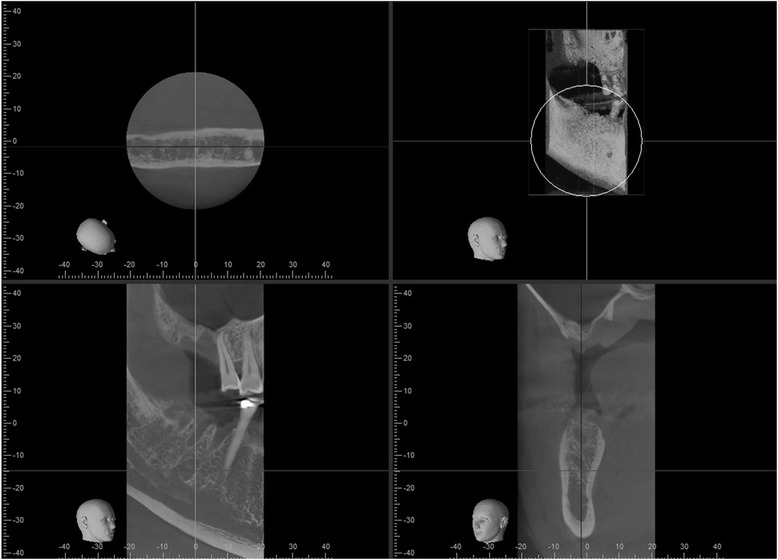
Right sinus prior to first sinus grafting procedure. Cone beam CT imaging shows very little residual bone volume at implant site for the no. 3 area

**Fig. 3:**
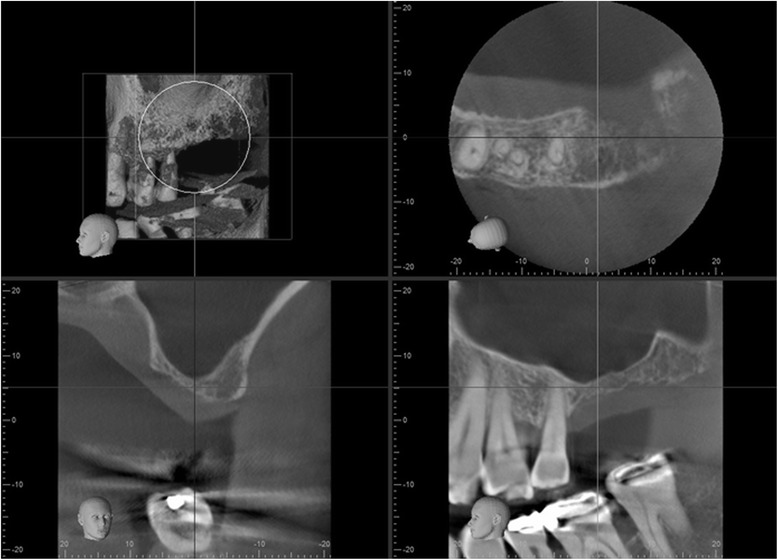
Left sinus prior to first sinus grafting procedure. Cone beam CT imaging also shows very little bone volume on left side for the no. 14 area

A 65-year-old retired Caucasian male presented to the Western University of Health Sciences Dental Center expressing an interest in implants after consulting with a private practice periodontist and a dentist from a large implant dentistry practice. He had no medical conditions or known allergies, but reported a 40-pack-year history of using tobacco and quit just before attending the Dental Center. No caries or mucosal abnormalities were found during examination other than a combined periodontal endodontic lesion at tooth no. 3 and localized severe periodontitis at no. 31 and no. 30 with complete through-and-through furcation involvement. Tooth no. 18 protruded beyond the occlusal plane, and several areas of shallow facial abfractions were noted on mandibular incisor teeth. (See initial panoramic radiograph, Fig. 1.) For initial disease treatment, teeth no.3, no. 30, and no. 31 were gently extracted and the residual socket of no. 30 grafted with human cortical particulate allograft. While healing was uneventful and ridge width was preserved at no. 30, little bone remained at the no. 3 site (see Fig. 2). On the left side, similar low amounts of available bone prevented implant placement at the no. 14 implant site (see Fig. 3). Given the good overall health of the patient, continued tobacco abstinence, good oral mucosal health after initial therapy, and low amount of sinus anatomy complexity, we suggested lateral window sinus augmentation to the patient, and the patient accepted proposed treatment after explanation of risks, benefits, and alternatives to implant therapy.

All of the following surgeries were carried out under local anesthesia. The patient received one tablet of 0.25 mg triazolam the evening before the surgery appointment and was taking ibuprofen 600 mg every 6 h and amoxicillin 250 mg every 6 h for 1 week starting the evening before the surgery. Starting the second day after surgery, the patient was instructed to rinse twice daily with 0.5 oz. of chlorhexidine gluconate for 30 s after oral hygiene, and the patient was seen at least once 7 days after each surgical procedure for postoperative care and oral hygiene instruction.

Lateral window sinus augmentation was performed on each side during appointments spaced 3 months apart, following the technique developed by Tatum in 1974. For each site, a midcrestal mucoperiosteal incision with buccal releases was created, and the lateral Schneiderian membrane of the maxillary sinus exposed through an ovoid window osteotomy of about 15 mm diameter. Osteotomy was performed using a piezotome (Piezotome 2, Acteon North America, and Mount Laurel, NJ, USA). Thereafter, the Schneiderian membrane was reflected away from the inferior floor of the sinus cavity with a mushroom-shaped Piezotome insert (Sinus surgery kit, Acteon North America, Mount Laurel, NJ, USA) and Sinus curettes (Sinus surgery curette kit, ACE Surgical Supply, Brockton, MA, USA) until the inferior most 15 mm of the medial wall was felt and seen. During both surgeries, we noticed small tears of 5 mm in the mid-portion of the mobilized Schneiderian membranes and repaired those by placing a double layer of 2 cm × 2 cm × 1.5 mm thick collagen tape (RCT, cut to shape, ACE Surgical Supply, Brockton, MA, USA) over the tears, which stabilized the membrane. We then placed a 1:1:1 mixture of cancellous and cortical allograft (AlloOss, ACE Surgical Supply, Brockton, MA, USA) and bovine xenograft (NuOss, ACE Surgical Supply, Brockton, MA, USA) into the space created between the former floor of the sinus cavity and collagen tape-covered Schneiderian membrane. Buccal access windows were then covered with a resorbable collagen membrane (resorbable collagen, ConFORM, ACE Surgical, Brockton, MA, USA) as suggested by Wallace and Froum [16], and the surgical site closed with continuous sutures (PTFE 3-0, Cytoplast, Osteogenics, Lubbock, TX, USA). No complications were reported by the patient and only when questioned he reported a short-lived episode of postnasal drip with few embedded “sand grains” after the surgery on the left side. We waited then for 10–12 months prior to further evaluation to allow complete dissolution of allograft [17] and allow complete bone formation [18].

**Fig. 4:**
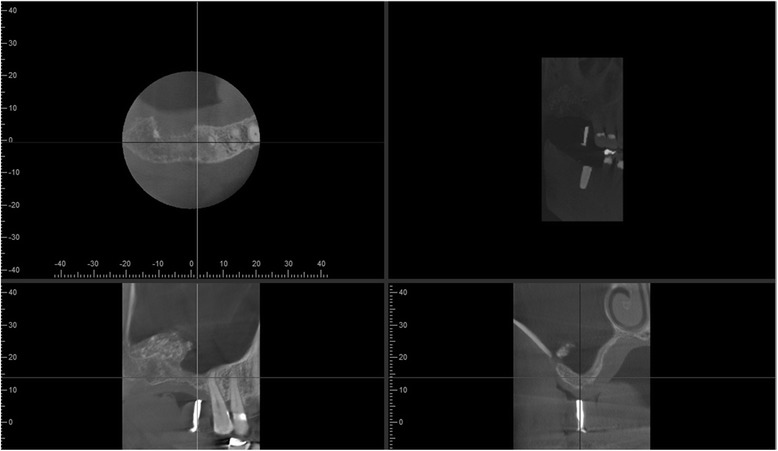
Right sinus about 12 months after first grafting procedure. Cone beam CT imaging shows little suitable bone at implant site, but grafted bone displaced distal to site. Bone hydroxyapatite particles were added as radiographic marker to the graft material for the first sinus augmentation procedure and are still visible as radiopaque specks

**Fig. 5:**
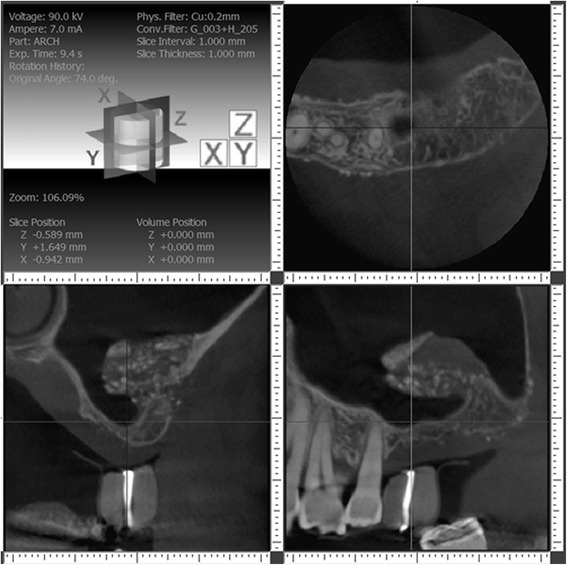
Left sinus about 12 months after first grafting procedure. Cone beam CT imaging shows unusual sinus anatomy after grafting, with finger-like sinus extension at implant site, and thick-grafted bone buccal and apical to it. The infractured wall is still clearly visible, as well as the bovine bone particles used as radiographic marker

A year later, we requested cone beam computed tomography for both posterior maxilla sites, and we found incomplete bone growth in the sinus. On the right side, bone growth had occurred only distal to the desired implant site, and there was an ovoid extension of sinus into the area planned for implant placement (Fig. 4). On the left side, a finger-like extension of sinus had developed between grafted bone and the former inferior medial wall of the sinus (Fig. 5). After explanation of findings, treatment alternatives, and risks and benefits of proposed treatments, the patient agreed on continuing with additional bone grafting.

**Fig. 6:**
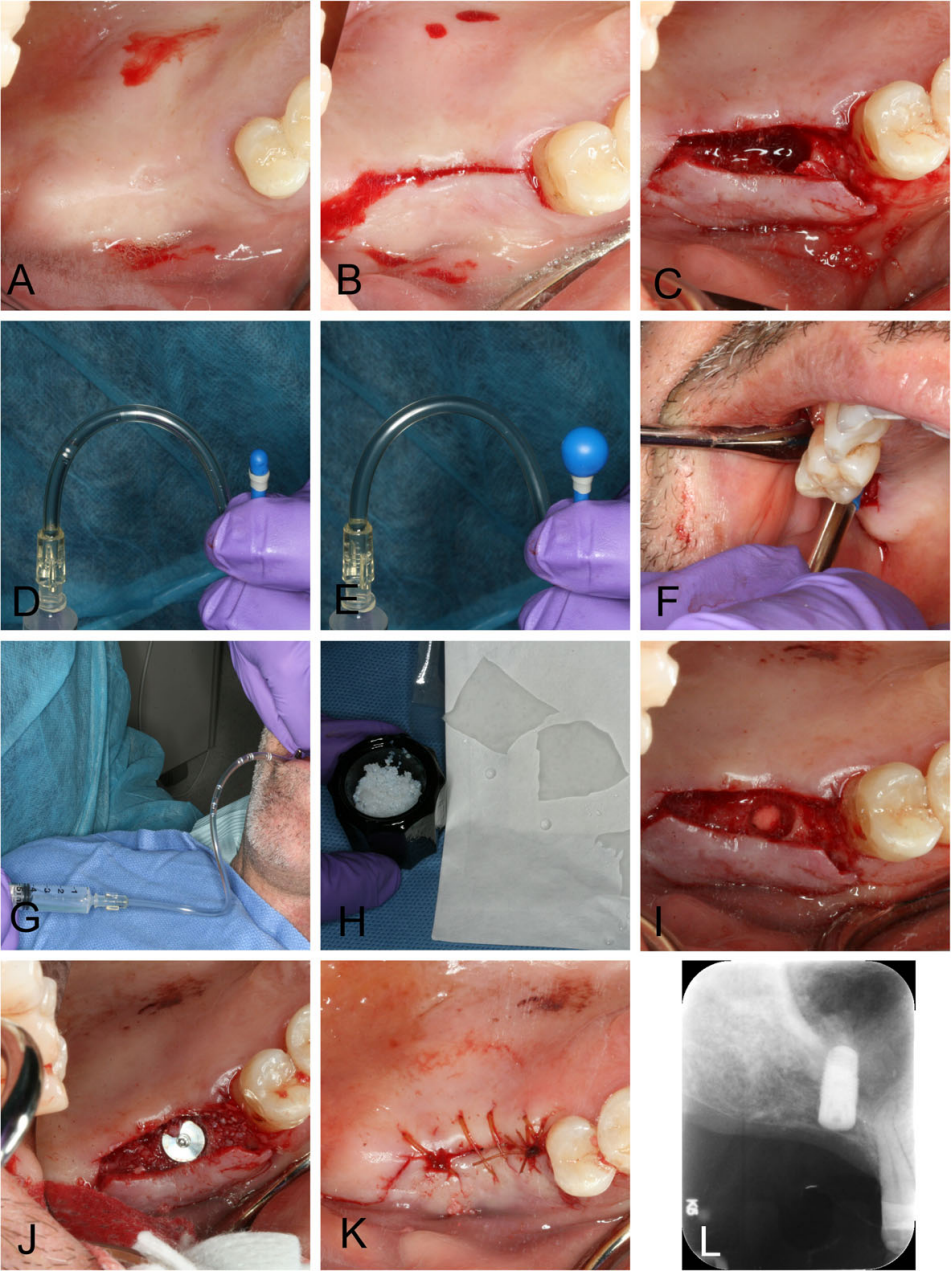
Right sinus balloon dilation procedure. This photographic series shows the surgical procedure that augmented bone and allowed implant placement at the no. 3 site. **a** Preoperative view after infiltration anesthesia. **b** Full-thickness midcrestal incision. **c** Osteotomy preparation with implant drills and osteotomes. **d, e** The dilating balloon, which is inflated using saline pressure from a syringe. **f** Insertion of uninflated balloon into osteotomy. **g** Gentle inflation of balloon by 1 ml. **h** Preparation of allograft and collagen tape. **i** Collagen tape is visible at bottom of osteotomy after filling expanded Schneiderian membrane with bone graft and covering graft with collagen tape. **j** Implant placement. **k** Suturing with a continuous suture. **l** Postoperative radiograph showing implant and halo of allograft surrounding apex of implant after surgery

**Fig. 7:**
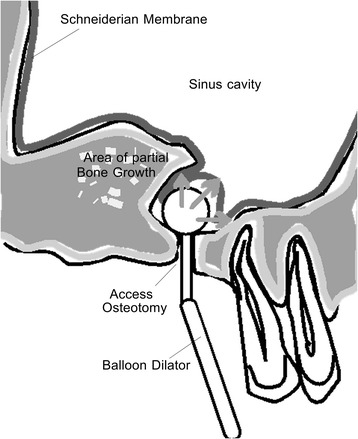
Schematic diagram of sinus balloon dilating procedure. This diagram shows how the balloon is inserted into a small transcrestal osteotomy and then expanded with balloon

For the right side, we decided to augment the area of insufficient bone using a balloon dilation technique through a subantral approach since the area of the missing bone was nearly spherical and centered at the no. 3 site. We also decided to place an implant simultaneously since primary stability seemed likely with the consistent thickness of 5 mm available bone at the no. 3 site, consistent with the recommendation by Pjetursson and Lang [19]. We created sinus access in a similar fashion as developed by Tatum in the 1970s and described by Misch [20] and performed sinus augmentation with a balloon technique as described for lateral window augmentation by Muronoi et al. [6]. (See Fig. 6 for the actual procedure, Fig. 7 for a diagram.) For this surgery, we created a mucoperiosteal flap with buccal releases for improved access (Fig. 6a, b) and created an osteotomy using osteotomy drills (Fig. 6c; Zimmer implant surgical kit, Zimmer, Carlsbad, CA, USA). Since there was sufficient ridge width and the bone was hard, we opted not to use Summer’s technique [21] but used drills to take the osteotomy to its final width that was slightly undersized for a 4.7-mm implant, but wide enough to allow insertion of a balloon dilator (straight model, Osseous Technologies of America, Hamburg, NY, USA). Drilling of the osteotomy stopped short 1 mm of the sinus floor. Prior to balloon dilation, we mobilized the Schneiderian membrane by gently infracturing small segments of the osteotomy floor using thin flat-ended osteotomes (ACE Surgical Supply, Brockton, MA, USA). For this, we started in the center of the osteotomy, advanced the depth of the infracture by 1 mm with a mallet and worked in a spiral fashion to the outer limits of the osteotomy floor and apical most 2 mm of the osteotomy wall. We then used a larger flat osteotome to advance the entire floor of the osteotomy by another millimeter, which resulted in a rubber-like mobility of the osteotomy floors. We verified the integrity of the membrane by gentle probing with a WHO probe and inserted the balloon dilator (Fig. 6d–g). We then slowly inflated the balloon dilator with 1 ml of saline, verified integrity of the membrane again, placed two sheets of 1 cm × 1 cm × 1.5 resorbable collagen tape, followed by 0.5 ml allograft and a 4.7 × 10 mm rootform implant (Fig. 6h–j; Tapered Screw-Vent TSVWB10, Zimmer, Carlsbad, CA, USA), which achieved good primary stability in excess of 30 Ncm. We placed a cover screw, replaced the flap, and sutured it with a continuous chromic gut 4-0 suture (Fig. 6k). Postoperative radiographs verified implant placement and showed good confinement of graft material around the implant (Fig. 6l). Healing was uneventful with only mild short-lived postoperative pain for a few days, and implant uncovery 12 months later revealed a firmly embedded implant.

**Fig. 8:**
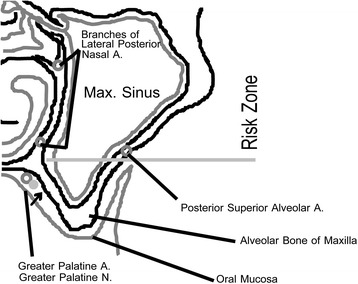
Blood supply of the sinus. There are three areas in the sinus where blood vessels may be encountered during sinus augmentation procedures for implants. On the inflection point between hard palate and alveolar ridge in the posterior maxilla, the greater palatine neurovascular bundle is located embedded in soft tissue. This inflection point is matched in the internal sinus anatomy and presents a landmark that can be palpated with sinus curettes during sinus membrane elevation or seen on cone beam CT images in this patient. It is important to avoid instrumenting the area above this inflection point as branches of the lateral posterior nasal arteries may be encountered superior to this area. Injuring these blood vessels can lead to significant sinus bleeding that is difficult to stop without sinus tamponade. Often on cone beam CT images, we see a small blood vessel channel midway within the lateral wall of the sinus, which likely is the posterior superior alveolar artery and vein. This and the interior medial wall sinus inflection point can serve as anatomic landmark to delineate a risk zone superior to it and to limit sinus augmentation inferior to it

**Fig. 9:**
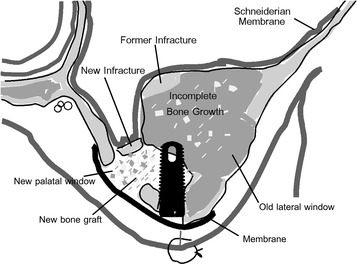
Schematic diagram of palatal approach sinus augmentation. The diagram shows the location of the lateral window, avoiding the thick grafted bone on the buccal, and the greater palatal neurovascular bundle

**Fig. 10:**
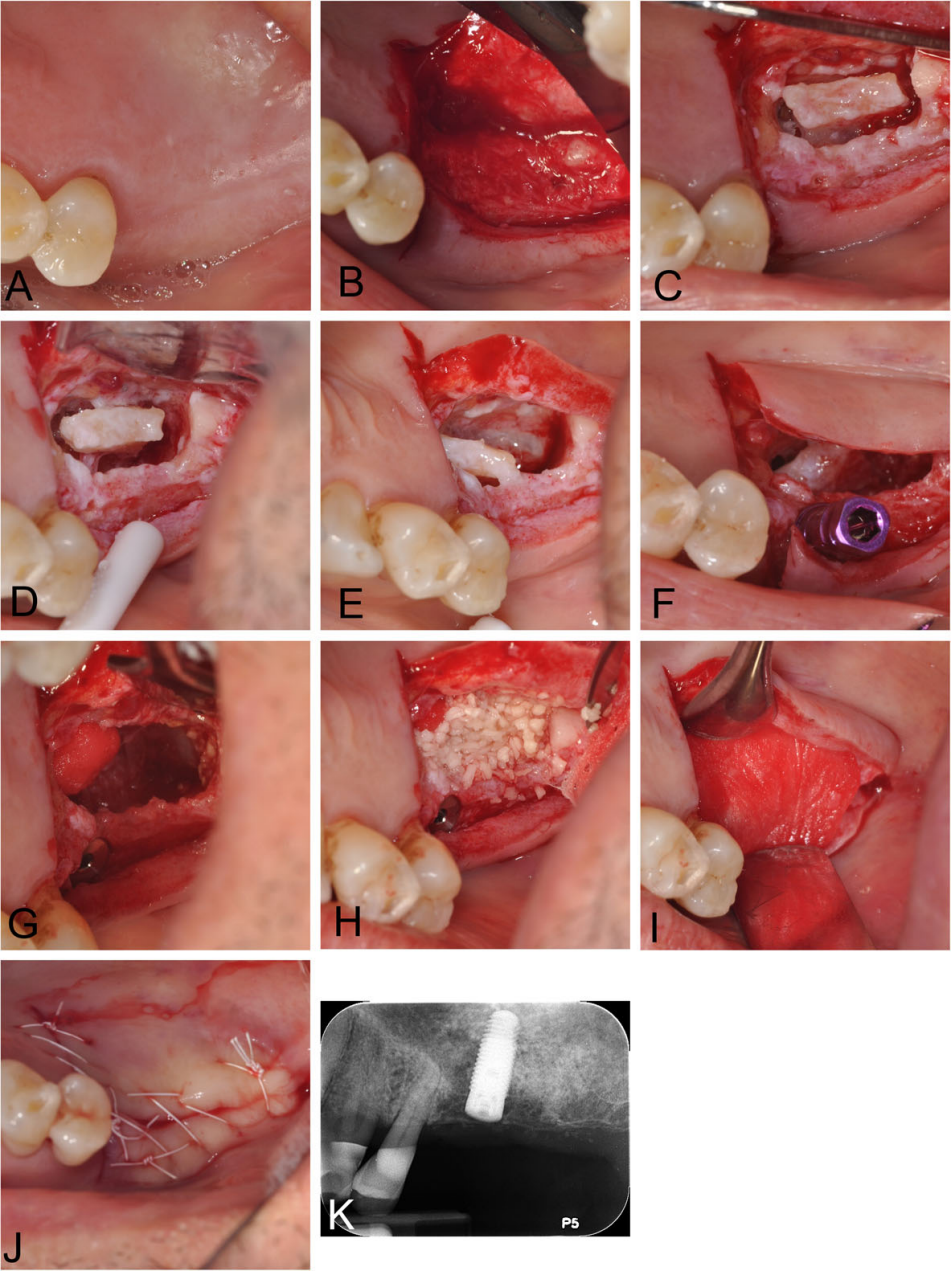
Palatal approach lateral window sinus augmentation. This photographic series shows the surgical procedure that augmented bone and allowed implant placement at the no. 14 site. **a** Preoperative view prior to infiltration anesthesia. **b** Full-thickness midcrestal incision with palatal release and flap elevation. This was aided by a small bony ridge that separated the alveolar crest from the soft tissue area containing the greater palatine neurovascular bundle. **c** Sinus window created with piezosurgery. **d–f** With gentle piezocision and water pressure, the finger-like membrane is slowly mobilized and collapsed towards the remainder of the sinus cavity. The overlying bone serves to form a new floor covering the base of the finger-like cavity. **f** Conventional implant placement using osteotomy drills. **g** Any exposed sinus membrane is covered with collagen tape. **h** Particulate mineralized allograft is placed into the newly created space. **i** A resorbable collagen membrane is placed over the access window. **j** Palatal tissue is sutured over implant and grafted site with mattress sutures. **k** Postoperative radiograph taken immediately after surgery shows cloud of particulate grafted bone around implant, suggesting good bone graft containment

For the left side, we decided to access the sinus using a lateral window as the area of deficient bone was much larger in size and more complex in shape. We also decided to approach this area from the palate, as the defect was closer to the palate and required much less bone removal as a buccal approach. Most importantly, we were already familiar with the anatomical structures on the lower medial wall of the sinus in the access area as we visualized this area during the first graft surgery and CT scans showed no signs of larger intraosseous vasculature in the area. Given this specific case, and knowledge of the vascular anatomy of the maxillary sinus in the surgical area (Fig. 8, based on CT scans of this patient and Bailey et al.’s work [22]), we felt that our approach would not invade the zone of risk for bleeding complications. We performed the surgery similar to a conventional lateral window sinus augmentation surgery using piezosurgery and a buccal approach, except from the palatal side of the alveolar ridge and staying clear of the greater palatine neurovascular bundle (Fig. 9). Here, we created a mucoperiosteal flap with vertical release at no. 13 (Fig. 10a, b). Using a piezotome and piezosurgery inserts (Piezotome 2, Acteon North America, Mount Laurel, NJ, USA), we created a rectangular window over the bony defect, avoiding any vascular structures (Fig. 10c). Using piezosurgery inserts and hydraulic pressure (IntraLift Kit, Acteon North America, Mount Laurel, NJ, USA), we carefully removed the Schneiderian membrane from the finger-like defect (Fig. 10d–f). We then placed a root-form 4.7 mm × 10 mm implant (Fig. 10f; Tapered Screw Vent TSWB10, Zimmer, Carlsbad, CA, USA) according to standard protocol and achieved good primary stability in excess of 30 Ncm. We placed a strip of resorbable collagen tape over any exposed Schneiderian membrane, grafted the site with 1.2 ml cortical particulate allograft (LifeNet Health, Virginia Beach, WA, USA) and placed a resorbable collagen membrane (ConFORM, ACE Surgical Supply, Brockton, MA, USA) over the palatal access window (Fig. 10g–i). We then covered the implant and graft with the palatal flap and sutured it with PTFE 3-0 (Cytoplast, Osteogenics Biomedical, Lubbock, TX, USA) continuous and horizontal mattress sutures (Fig. 10j). A postoperative radiograph showed good containment of the graft material (Fig. 10k).

**Fig. 11:**
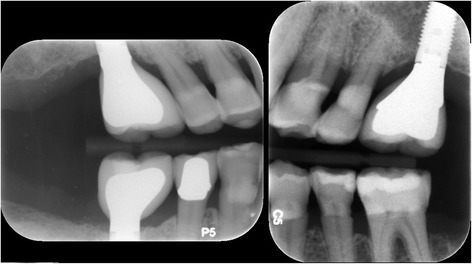
Implant restoration. Implants were restored by dental students supervised by prosthodontists at the Dental Center

**Fig. 12:**
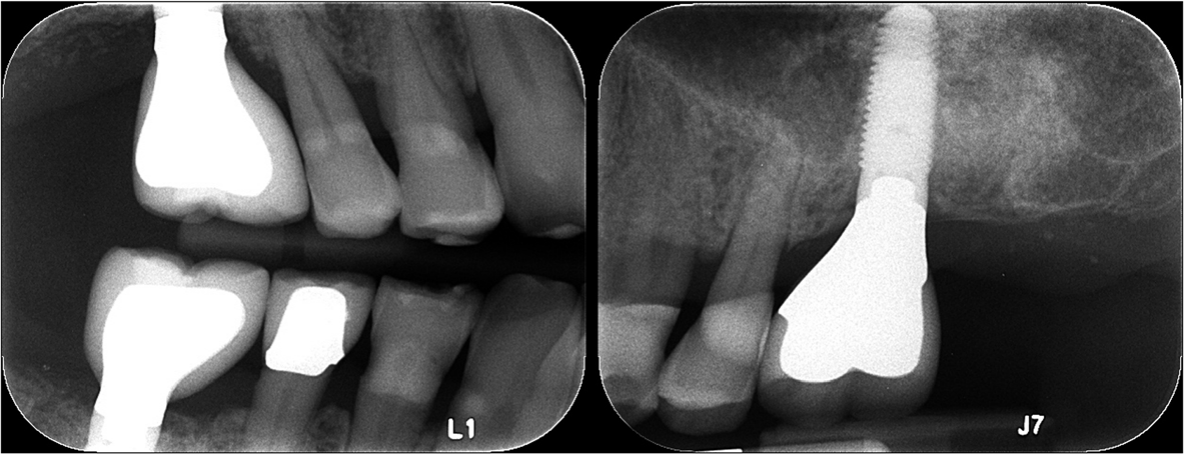
Radiographic bone levels three years after placement. Bone levels remain unchanged during long-term follow-up

Healing was uneventful with little discomfort reported by the patient during the first week, and implant uncovery revealed an implant firmly embedded in bone after 12 months. A third implant was placed at the no. 30 site and supraerupted no. 18 extracted as planned. Restoration of the implants was uneventfully performed by senior dental students supervised by various prosthodontists (Fig. 11). Periodontal maintenance was regularly performed, and 3 years after implant placement, there is no significant bone loss (Fig. 12), and probing depth remains at 2 to 4 mm with no bleeding on probing.

## Conclusions

We conclude that incomplete bone formation after sinus augmentation can be managed successfully through a variety of re-entry procedures and that successful long-term implant placement and restoration is possible in a compliant patient of good overall health.
